# P-1505. Impact of the Introduction of Ceftazidime-Avibactam in the Brazilian Market on Carbapenem and Ceftazidime-Avibactam Resistance in Klebsiella pneumoniae and Pseudomonas aeruginosa: Retrospective Analysis from 2017 to 2023

**DOI:** 10.1093/ofid/ofae631.1674

**Published:** 2025-01-29

**Authors:** Matias C Salomão, Maristela P Freire, Nathamy F dos Santos, Karin M Macedo, José De Sá, Paola Cappellano

**Affiliations:** Grupo Fleury, São Paulo, Sao Paulo, Brazil; Hospital das Clínicas da Faculdade de Medicina da Universidade de São Paulo, São Paulo, Sao Paulo, Brazil; Grupo Fleury, São Paulo, Sao Paulo, Brazil; Grupo Fleury, São Paulo, Sao Paulo, Brazil; Grupo Fleury, São Paulo, Sao Paulo, Brazil; Grupo Fleury, São Paulo, Sao Paulo, Brazil

## Abstract

**Background:**

Bacterial resistance to carbapenems, poses a global health challenge, with *Klebsiella pneumoniae* and *Pseudomonas aeruginosa* as significant contributors. Ceftazidime-avibactam (CZA), introduced in Brazil in June 2018, offers a new treatment option for multi-resistant infections. However, increasing resistance to both this drug and carbapenems raises concerns.

Our goal is to evaluate the impact of CZA introduction on resistance patterns, and metallo-betalactamases (MBL) prevalence in Brazil, focusing on *K. pneumoniae* and P. *aeruginosa*.

**Figure:**
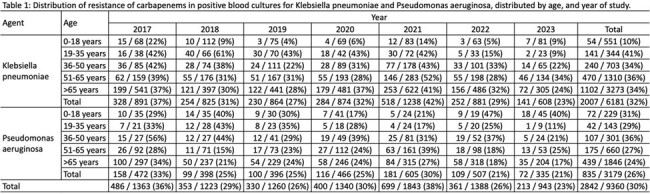

**Methods:**

A retrospective analysis of microbiology data from a Private Laboratory (2017-2023) was conducted. Samples from 9 Brazilian states were assessed. We included all positive blood cultures and all resistant surveillance swab cultures for *K. pneumoniae* and *P. aeruginosa*. We excluded repeated positive blood cultures in a week interval with the same agent, and we considered only the first positive resistant surveillance culture.

We considered carbapenem-resistant if the isolate was resistant to any carbapenem. It was evaluated phenotypically or genotypically and carbapenemase production patterns were aggregated into 3 categories: serine-, MBL, or co-production. We used Poisson regression model with a robust error variances variance to compare pre- and post-CZA introduction resistance prevalences. The models were adjusted for COVID-19 national incidence, age, and gender.

**Figure:**
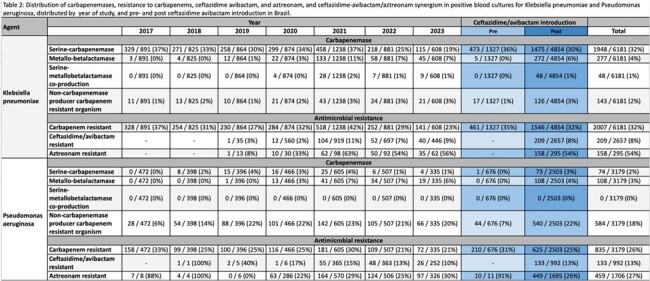

**Results:**

We analyzed 9361 positive blood and 16862 carbapenem-resistant surveillance cultures. In blood cultures, CZA resistance rose after its introduction in *K. pneumoniae* (3% to 9%; 2019 to 2023 (p < 0.001), so did the MBL production (0% to 6%, p 0.02), and serine- and MBL co-production: 0% to 1% (p 0.04). MBL also increased in *P. aeruginosa* during the study period: 0% to 4% from 2017 to 2023 (p < 0.001).

In surveillance cultures, carbapenemase production increased in *P. aeruginosa* after CZA introduction: 5% to 22%, p < 0.001.

**Figure:**
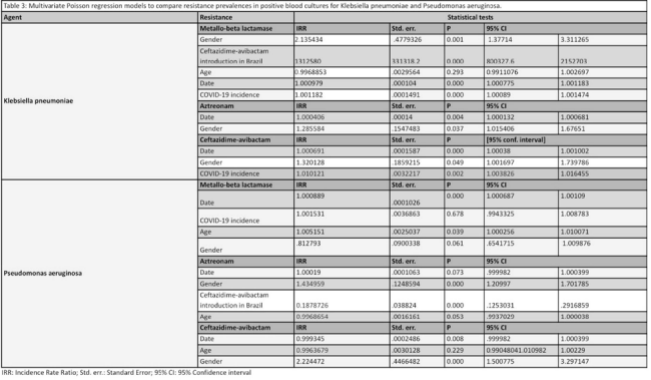

**Conclusion:**

The observed increases in resistance prevalence and carbapenemase production associated with CZA introduction indicate limitations for its use. Enhanced surveillance and antimicrobial stewardship strategies are urgently needed to address the growing threat of antimicrobial resistance.

**Disclosures:**

**All Authors**: No reported disclosures

